# Dynamics of nuclear matrix attachment regions during 5^th^ instar posterior silk gland development in *Bombyx mori*

**DOI:** 10.1186/s12864-022-08446-3

**Published:** 2022-03-31

**Authors:** Alekhya Rani Chunduri, Resma Rajan, Anugata Lima, Senthilkumar Ramamoorthy, Anitha Mamillapalli

**Affiliations:** 1 Department of Biotechnology, Institute of Science, GITAM (Deemed to be University), Visakhapatnam, Andhra Pradesh 530 045 India; 2 Institute of Medical Bioinformatics and Systems Medicine, Medical Center, Faculty of Medicine, University of Freiburg, 79110 Freiburg im Breisgau, Baden-Wüttemberg Germany; 3 Division of Pediatric Hematology and Oncology, Department of Pediatrics and Adolescent Medicine, University of Freiburg, 79106 Freiburg im Breisgau, Baden-Wüttemberg Germany

**Keywords:** Nuclear matrix, Matrix attachment regions, *Bombyx mori*, Posterior silk glands, Fibroin

## Abstract

**Background:**

Chromatin architecture is critical for gene expression during development. Matrix attachment regions (MARs) control and regulate chromatin dynamics. The position of MARs in the genome determines the expression of genes in the organism. In this study, we set out to elucidate how MARs temporally regulate the expression of the fibroin heavy chain (FIBH) gene during development. We addressed this by identifying MARs and studying their distribution and differentiation, in the posterior silk glands of *Bombyx mori* during 5th instar development.

**Results:**

Of the MARs identified on three different days, 7.15% MARs were common to all 3 days, whereas, 1.41, 19.27 and 52.47% MARs were unique to day 1, day 5, and day 7, respectively highlighting the dynamic nature of the matrix associated DNA. The average chromatin loop length based on the chromosome wise distribution of MARs and the distances between these MAR regions decreased from day 1 (253.91 kb) to day 5 (73.54 kb) to day 7 (39.19 kb). Further significant changes in the MARs in the vicinity of the FIBH gene were found during different days of 5^th^ instar development which implied their role in the regulation and expression of the FIBH gene.

**Conclusions:**

The presence of MARs in the flanking regions of genes found to exhibit differential expression during 5^th^ instar development indicates their possible role in the regulation of their expression. This reiterates the importance of MARs in the genomic functioning as regulators of the molecular mechanisms in the nucleus. This is the first study that takes into account the tissue specific genome-wide MAR association and the potential role of these MARs in developmentally regulated gene expression. The current study lays a foundation to understand the genome wide regulation of chromatin during development.

**Supplementary Information:**

The online version contains supplementary material available at 10.1186/s12864-022-08446-3.

## Background

The role of the nuclear matrix (NuMat) in the spatial and temporal organization of the genome makes it especially significant in DNA replication, gene expression, and regulation [1–3]. The NuMat facilitates the arrangement of chromatin into loop structures by anchoring it to the nucleus [4]. These DNA sequences anchor the chromatin loops and are known as scaffold or matrix attached regions (MARs) and therefore their dynamic nature is essential to the study of chromatin architecture and the organisation of the genome [5]. MARs are often correlated with chromatin features like, AT richness, the origin of replication, etc., and are also hotspots for TE insertions [6]. Many studies have been carried out determining their role in the maintenance of chromatin architecture, stabilization and regulation of gene expression [7–9] and their influence on gene silencing mechanisms [10–12].

The whole genome sequencing of MARs has been carried out by very few studies. A genome-wide analysis of MARs belonging to the euchromatic part of the genome was performed in *Drosophila melanogaster* and identified 7353 MARs [13]. The MAR DNA isolated from embryos of *D. melanogaster* identified the LTR (Long terminal repeat) TE ‘*roo’* which showed high similarity with the TE *gypsy* [14]. In *Arabidopsis*, the genome-wide in-silico MAR identification showed a significant correlation between the presence of intragenic S/MARs and transcriptional down-regulation [15]. Several studies were also carried out to investigate MARs in vertebrates such as humans, mice, chickens and Chinese hamsters [16–19]. The influence of MARs in gene expression and their successful integration and regulation of transgenic genes were also determined [19–22].

The silkworm, *Bombyx mori (B. mori)* is a widely commercialized insect that is agriculturally and economically relevant. The silk produced by this insect has extensive industrial and medical applications [23]. The silk gland is a complex organ that is known for its tissue-specific gene expression and regulation during development [24]. The silk glands were found to show an increase in the genomic DNA content by 200–300 times during larval development due to endomitosis [25]. The silk glands exhibit a significant and sudden growth during larval development. It was determined that the silkworm, *B. mori* has significantly more cells in its PSGs (posterior silk glands) that result in higher fibroin production when compared to the wild type silkworm due to the up-regulation of genes responsible for division and growth of cells which is attributed to its history of domestication [26].The compartmentalisation of the silk gland into different regions i.e., ASGs (anterior silk glands), MSGs (Middle silk glands) and PSGs is attributed to the Hox genes and their role in the regulation of expression in silk related genes [27]. The PSG synthesizes the most important protein involved in silk synthesis, Fib-H [28]. The spatial organization of chromatin in the multi-nucleated cells of silk glands is facilitated by the existence of the NuMat [29].

The FIBH gene which produces fibroin heavy chain protein is regulated by multiple factors. DNA sequences upstream of the gene were shown to possess transcription modulation signals [13, 30]. The 62 kb upstream region of FIBH, Bmmar1, a mariner like element previously identified in *Drosophila* and other insects, as well as Bm1 (a SINE element belonging to tRNA superfamily) and L1Bm (a LINE (long interspersed nuclear element) within the intronic region of FIBH) were found in the ORF regions [24, 31–34]. BmMar1 was found to specifically attach to the NuMat in silk glands and is predicted to be involved in the regulation of its expression, The core region of this MAR consisted of two degenerate sequences derived from L1Bm and Bm1 [30, 35]. The genome-wide MARs of silk glands and their distribution and differentiation in occurrence and positions during development has not been elucidated.

The current study investigates the genome-wide temporal regulation of MARs in PSGs of *B. mori* on different days of 5^th^ instar larval development. The presence of MARs is explored in the context of their possible role in the expression of FIBH. Chromosome-wide distribution and density of MARs were dynamic during the 5^th^ instar development. MARs varied in number and position flanking FIBH. Many MAR regions identified in the flanking regions of FIBH were found to be developmentally regulated.

## Results

### Ultrastructural analysis of silk gland NuMat reveals a fibro-granular network

**Fig. 1:**
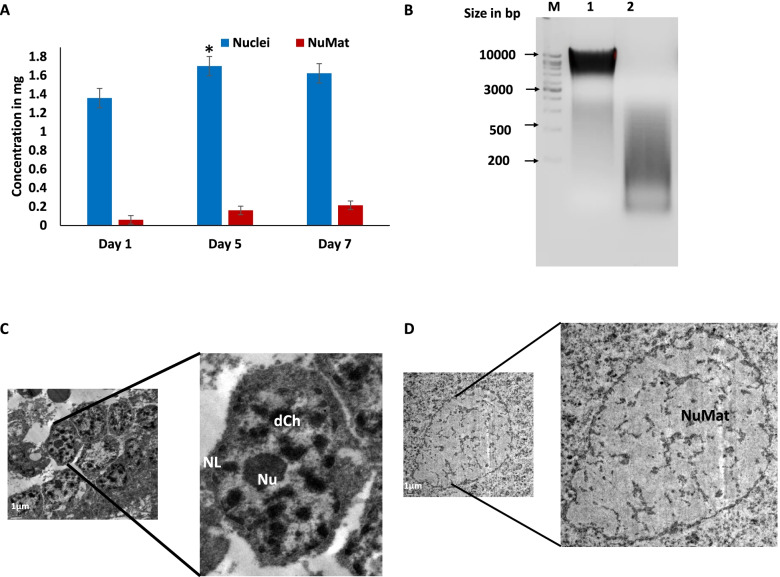
Isolation of MAR DNA from 5^th^ instar posterior silk glands of *B. mori.* **A** The nuclear and MAR DNA of day 1, day 5 and day 7 were quantified using U.V spectrophotometry (*n* = 5, where n is the number of sample replicates). Student’s T-test (two sample using unequal variances) used for statistical analysis and no significant enrichment was found in MAR DNA from day 1 to day 7 was observed (*p* > 0.05) while significant difference was observed between concentrations of nuclei and nuclear matrix (NuMat) DNA (*p* < 0.005). The nuclear DNA increased significantly from day 1 to day 5 but no significant increase was found on day 7. **B** A 1 kb DNA ladder (labelled ‘M’), the isolated nuclear DNA and MAR DNA from 5^th^ instar day 5 PSGs were run on lane 1, lane 2 and lane 3 respectively on a 1.2% agarose gel. **C** Transmission electron microscopy imaging of nucleus (left) isolated from day 5 of 5^th^ instar larvae, Scale: 1 μm, showing nucleolus (Nu), peripheral condensed chromatin (dCh) and nuclear lamina (NL) and **D** Transmission electron microscopy image of NuMat (right) isolated from day 5 of 5^th^ instar larvae revealed the continuation of the inner network towards the inner periphery of the nuclear lamina. The residual nucleolus was not observed in the NuMat preparation of this study. Scale: 1 μm. A contrasting line has drawn below the existing scale bar of both Fig. 1 (**C**) and (**D**) and the scale is also indicated above the original scale in contrasting colour for better visibility

The association of DNA with the NuMat is transient and is highly regulated during development to control gene expression. PSGs in which FIBH is specifically expressed, have been shown to exhibit large variations in the gene expression during different days of 5^th^ instar development [46]. The NuMat is isolated on day 1, day 5 and day 7 of 5^th^ instar from PSGs and DNA extraction is carried out from the NuMat preparations. The quantification of this DNA (MAR DNA) along with nuclear DNA is carried out to understand its nuclear retention in the NuMat on different days of 5^th^ instar stage. The results showed that the % retention of DNA from nucleus in NuMat is 4.33, 9.41 and 13.24% on day 1, day 5 and day 7 respectively (Fig. 1A). The agarose gel electrophoresis of MAR DNA showed its size to be in the range of 200-1000 bp (Fig. 1B). The nuclear and NuMat preparations of day 5 PSGs of 5^th^ instar stage were used for TEM (Transmission electron microscopy) imaging.

The ultrastructure of the NuMat reveals the internal architecture and confirms the depletion of chromatin during isolation. The nuclease digestion and high salt extraction revealed the inner nuclear skeleton. The PSG nucleus showed three well-defined compartments: the lamina (NL), inter-chromatin network, and a densely stained nucleolus (Nu). Condensed chromatin (dCh) was observed surrounding the nucleolus and lining the inner periphery of the nuclear lamina. In addition, condensed (dCh) and open chromatin (lCh) were observed as dark and light areas throughout the nucleus (Fig. 1C). The chromatin-depleted NuMat of PSG showed a fibro-granular network spanning across the nucleus. A distinct decrease in the condensed chromatin regions was observed. Many domains retained their positions after the removal of the soluble proteins, and chromatin; and correspond to structures that can be observed by electron microscopy in unfractionated nuclei and NuMat preparations; highlighting the importance of electron microscopic studies [47, 48]. The NuMat preparation still retained a small portion of chromatin along the inner periphery of the nuclear envelope. The residual nucleolus was not observed in the NuMat preparation (Fig. 1D). This result validated the procedure employed for NuMat isolation. A resinless section electron microscopy study showed the NuMat to consist of the nuclear lamina (NL) and an internal matrix connected to the lamina. This internal matrix is a network of irregular fibers with intricate fine structures evident in the images obtained.

### The genome-wide MAR profile of 5^th^ instar development

**Fig. 2:**
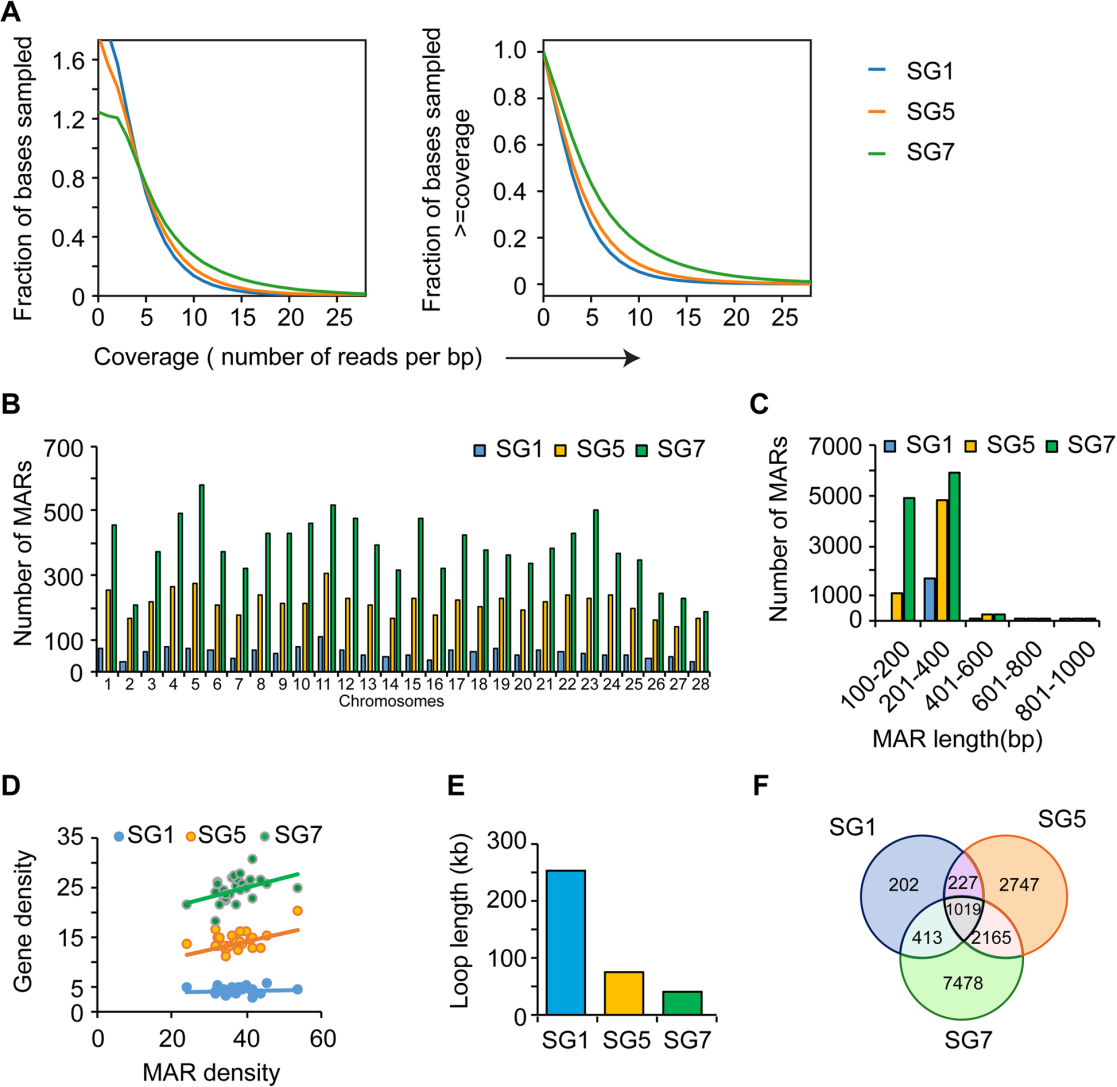
Analysis of Chromosome – wise distributed MARs. The MAR DNA from posterior silk glands extracted from day 1, day 5 and day 7 were sequenced using Illumina platform and mapped against the *Bombyx mori* annotated genome downloaded from Silkbase. **A** The depth (left) and coverage (right) of the mapped sequenced reads was analysed using PlotCoverage. **B** Chromosome wise distribution of MARs is shown. **C** Lengths of the MARs in the three datasets were derived from the enriched MAR peaks data. **D** Scatter plot of the density of MARs plotted against the gene densities for each chromosome is shown. **E** The enrichment of MAR regions on each chromosome was obtained using MACS2 callpeak. Chromatin loop length for each chromosome was obtained by calculating the distance between the MAR regions and the average loop length in each dataset is depicted in the graph. **F** Common and unique MARs among the three datasets are shown. SG1 and SG5 consist of less number of MARs. They are rich in repetitive regions. The number of MARs and unique MARs are more in SG7

The MAR DNA extracted from the three datasets was sequenced to analyse their genome-wide association. Mapped datasets obtained by next-generation sequencing techniques were analysed for coverage and sequencing depth prior to downstream analysis. Our analysis confirms the uniform coverage of mapped reads across the genome and indicates an equal number of reads aligned to a reference base position. The quality of the mapped reads in PSGs from day 1, day 5, and day 7 were evaluated. Results showed that 12% of the regions sampled were covered at least twice and 10% of the regions were covered at least 5 times. Mean values of SG 1, SG 5, and SG 7 (day 1 MAR dataset of 5^th^ instar PSGs, day 5 MAR dataset of 5^th^ instar PSGs, day 7 MAR dataset of 5^th^ instar PSGs respectively) were 4.0, 4.6, and 7.2, respectively (Fig. 2A, left). The data showed that 20% of the sampled base pairs had up to 10 overlapping reads in terms of sequencing depth, and 5% of the sampled base pairs exhibited more than 40 overlapping reads (Fig. 2A, right). Lower number of fraction of bases were observed in SG7. In our MARSeq datasets, we found relatively low abundance of regions with low coverage in SG7. This indicates high number of MARs in the SG7 dataset. We applied a very stringent approach to avoid reads from non-MAR regions. The regions with high read coverage were identified using the MACS2. Analysis of the raw sequenced data revealed an average of 74.85% unique sequences among the SG1, SG5, and SG7 datasets (Supplementary Table 1). Thus the obtained datasets from SG1, SG5, and SG7 were deemed good for further analysis.

### MAR dynamics in chromosome-wise distribution and developmental progression

The *B. mori* genome is 460.3 Mb in size and is organized in 28 chromosomes with each chromosome harbouring many genes [38]. The chromosome-wise distribution of MARs and MAR density was performed using the merged peaks derived from the mapped datasets, as it is important in understanding the gene expression of chromosomes [18, 49] (Fig. 2B). This analysis also helps us understand the topological association of MARs on the chromosomes. The highest MAR density in PSGs was detected at different days of 5^th^ instar development, on chromosomes 19, 2, and 5 on day 1, day 5, and day 7, respectively (Table 1). The MAR DNA sequences associated with the NuMat in the 200–1000 bp were found to be even more enriched in the size range of 200–400 bp (Fig. 2C). The MAR density has been shown to positively correlate with gene density (Fig. 2D). MAR density on chromosomes predicts chromatin loop size [18]. The increase in MAR density from SG 1 to SG 5 and SG 5 to SG 7 showed decreased chromatin loop size and an increase in the number of chromatin loops as the development of 5^th^ instar *B. mori* progressed (Fig. 2E). The distribution of MARs determines the chromatin loop formation and is thus imperative in understanding the gene regulation processes [50]. The average chromatin loop length decreased from day 1 (253.91 kb) to day 5 (73.54 kb) to day 7 (39.19 kb). A weak positive correlation was found between MAR count and gene density in SG 1, SG 5, and SG 7 datasets (Supplementary Fig. 1). These findings showed that chromosome-wise differences and developmental differences (day-wise) exist in MAR association with the NuMat.

**Table 1:** Chromosome-wise distribution of MARs. The number of MARs per chromosome were identified and their density was calculated (number of MARs/ Size of Chromosome)

Chromosome	Size (Mb)	No. of genes	Gene density	MAR count			MAR/Mb		
				SG1	SG5	SG7	SG1	SG5	SG7
1	20.66	710	34.37	73	255	454	3.53	12.34	21.97
2	8.39	452	53.87	35	168	206	4.17	20.02	24.55
3	15.21	569	37.41	63	216	375	4.14	14.20	24.65
4	18.73	769	41.06	78	263	490	4.16	14.04	26.16
5	19.06	798	41.87	76	275	577	3.99	14.43	30.27
6	16.65	568	34.11	69	209	374	4.14	12.55	22.46
7	13.94	489	35.08	46	176	320	3.30	12.63	22.96
8	16.26	586	36.04	71	239	430	4.37	14.70	26.45
9	16.79	542	32.28	60	215	432	3.57	12.81	25.73
10	17.61	670	38.05	77	212	461	4.37	12.04	26.18
11	20.44	928	45.40	110	304	515	5.38	14.87	25.20
12	17.58	651	37.03	68	228	476	3.87	12.97	27.08
13	17.73	614	34.63	56	209	394	3.16	11.79	22.22
14	13.34	427	32.01	48	168	314	3.60	12.59	23.54
15	18.44	807	43.76	56	228	477	3.04	12.36	25.87
16	14.33	600	41.87	36	175	320	2.51	12.21	22.33
17	16.84	662	39.31	70	225	427	4.16	13.36	25.36
18	15.69	538	34.29	66	201	378	4.21	12.81	24.09
19	14.80	592	40.00	73	229	361	4.93	15.47	24.39
20	12.37	474	38.32	54	195	339	4.37	15.76	27.41
21	15.31	499	32.59	71	219	384	4.64	14.30	25.08
22	18.48	659	35.66	63	238	430	3.41	12.88	23.27
23	21.46	739	34.44	61	230	501	2.84	10.72	23.35
24	17.35	646	37.23	56	238	369	3.23	13.72	21.27
25	14.54	560	38.51	56	196	349	3.85	13.48	24.00
26	11.47	378	32.96	45	164	242	3.92	14.30	21.10
27	10.93	264	24.15	47	143	230	4.30	13.08	21.04
28	10.60	337	31.79	33	169	190	3.11	15.94	17.92

### Increased abundance of MARs during 5^th^ instar development

After the quantitative MAR DNA analysis of the three developmental time points (day1, day 5, and day 7), we looked into the number of MARs present in the sequenced NuMat extractions in each of the datasets (SG1, SG5, and SG7). The number of MARs in each dataset was identified by peak calling on the mapped reads. The results showed a total of 1861, 6158, and 11,075 MAR regions in SG1, SG5, and SG7, respectively. To understand the dynamics of MARs during 5^th^ instar development, the common and unique regions were identified in three datasets. 1019 MAR regions were found to be common among SG1, SG5, and SG7 (Fig. 2F). The number of unique MARs increases from day 1 to day 7 suggesting the role of these MARs in the expression and regulation of genes involved in silk gland development. 7.15% MARs were found to be common to all 3 days and 1.41, 19.27, and 52.47% MARs were unique to day 1, day 5, and day 7, respectively. The unique sequences may hold clues in further understanding the development-based differential gene expression of PSGs.

### Short tandem repeats- and TEs are associated with PSG NuMat

**Fig. 3:**
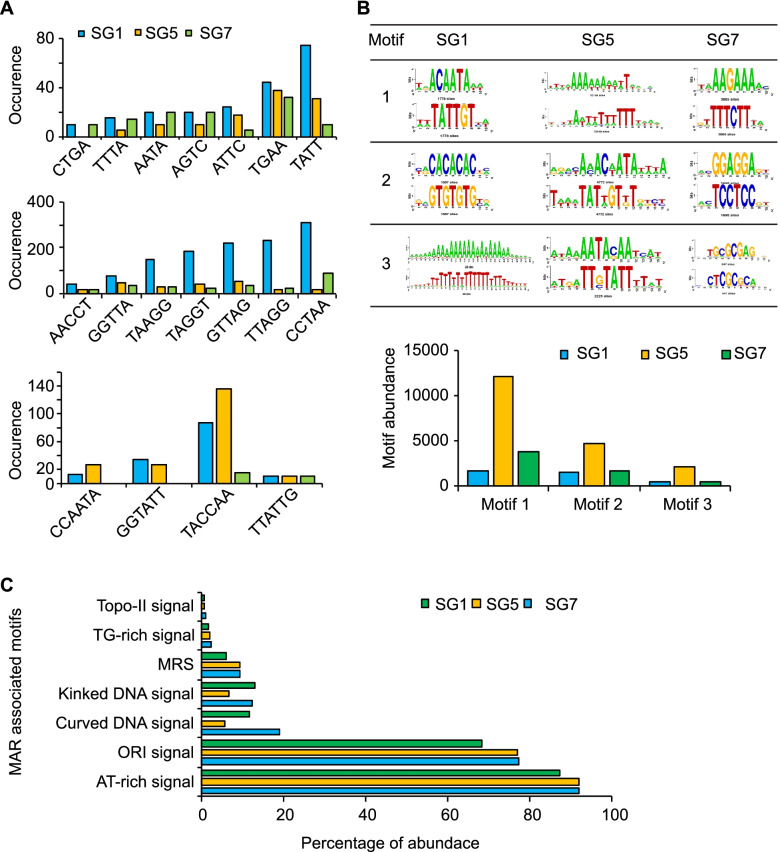
Enrichment of Simple tandem repeats and motifs. **A** Tetra, penta, hexa--nucleotide repeats enriched in the datasets. The y-axis shows the occurrence of these repeat signals in the datasets and the x-axis shows the repeat sequences. **B** The three most abundantly occurring motifs in the three datasets (above) and their abundance (below). The y-axis shows the number of sequences detected with reference to the specific motifs and the x-axis represents the specific motifs that were found in abundance in the datasets. **C** Percentage abundance of each the seven MAR associated motifs in the datasets

Several repeat sequences are associated with the NuMat [51, 52], and STRs (Short tandem repeats) in particular play an important role in gene identification [53]. Hence, STR analysis was performed to understand their association with the NuMat during PSG development. We found an enrichment of STRs in SG 1 compared to SG 5 and SG 7. TATT, TGAA, and AGTC tetranucleotides; CCTAA, TTAGG, and GTTAG pentanucleotides; and TACCAA, and TTATTG hexanucleotides were the most abundant (Fig. 3A). The MARs of SG 1, SG 5, and SG 7 showed AT- richness suggesting a high association of functional genes with the matrix in PSGs.

Several studies demonstrate the relationship between transposable elements (TEs) and gene expression [54, 55]. The TEs associated with the NuMat provide necessary spatial arrangement of promoters and enhancers and aid the binding of MARs with MAR binding proteins. Many regulatory regions are associated with TEs, as indicated by the whole-genome analyses of *Caenorhabditis elegans* [56], *Schizosaccharomyces pombe* [57], and humans [58, 59]. The retrotransposon sequences of LTRs and LINE elements are enriched in MARs of the *Drosophila* euchromatic genome [13]. RepeatMasker analysis revealed LINEs, LTR elements, and other repeats association with the NuMat of PSG. The largest number of LINE elements and LTR elements was found on day 7. While the LINE elements occupied a total of 4526 bp, 427 bp, and 22,760 bp in SG 1, SG 5, and SG 7, respectively; the LTR elements occupied 111 bp and 430 bp in SG 5 and SG 7, respectively (Table 2). LINEs have internal promoters that initiate transcription upstream of the 5′end of the element or are co-transcribed along with the target site from an external host promoter. The enrichment of MARs in LINE elements on day 7 validates their role in the expression of genes related to the final stage of larval development and silk production. The TE L1Bm (a LINE element), was shown to be matrix-associated. It is located in the upstream region of the FIBH promoter and was previously predicted to be involved in the regulation of the FIBH gene [34, 35]. L1Bm was attached to the NuMat of PSGs on all 3 days of 5^th^ instar larval development. R1Bmks, another LINE element, was found to have regions of association with the NuMat on day 1 and day 7 but not on day 5. Thus, the differences in the enrichment and distribution of STRs and TEs in the PSG developmental datasets hint at the complexity and multi-factorial regulation of the development associated gene.

**Table 2:** TEs associated with posterior silk gland NuMat: Repeat Masker was used with rmblast by masking interspersed and simple repeats against Drosophila TE database. L1Bm and R1Bmks were identified in the three datasets using BLAST2 (hits with > = 90% percent identity and > =100 bp were considered)

S. No.	Type	Number of elements			Length occupied (bp)			Percentage of sequence (% )		
		SG 1	SG 5	SG 7	SG 1	SG 5	SG 7	SG 1	SG 5	SG 7
1.	LINEs									
	L1Bm	12	2	84	3108	427	20512	0.56	0.0261	0.7997
	R1Bmks	2	-	2	1418	-	2124	0.256		0.082
	R1/LOA/Jockey	0	0	2	0	0	124	0.00	0.00	0.00
2	LTR elements									
	Ty1/Copia	0		2	0		262	0.00		0.01
	Gypsy/DIRS1	0	1	1	0	111	168	0.00	0.01	0.01
3	Total interspersed repeats:	-	-	-	1360	196	11060	0.25	0.01	0.43
4	Small RNA	39	1	43	12045	107	12993	2.18	0.01	0.51
5	Simple repeats	396	877	745	24797	55758	48261	4.48	3.41	1.88
6	Low complexity	61	209	153	3317	10250	7744	0.60	0.63	0.30

### An evolutionarily conserved sequence features in the PSG MARs

The distribution of certain motifs in the NuMat correlates with gene expression [60–62]. The role of transcription factors (TFs) and their binding to DNA sequences such as the MAR regions and the regulatory elements: promoters, enhancers, ORIs, and silencers, to aid the regulation of gene expression in eukaryotes has also been explored by previous studies [63]. The use of MARs in transfected cells, regardless of species, demonstrated their role in epigenetic maintenance and consistent insulating activity that allows for relatively stable gene expression [20, 21]. MARs were found to enhance the transgene expression and the MAR assisted transcriptional activation of these transgenes involved AT-rich sequences and specific TF binding motifs [22]. The 5^th^ instar developmental datasets of SG 1, SG 5, and SG 7 were scanned for the presence of the most abundantly overrepresented DNA motifs, which are most often TF binding sites. The motif patterns in SG1, SG5, and SG7 were found to be different (Fig. 3B). The MAR-associated motifs such as ORI signals, topoisomerase II signals, TG rich signal, AT-rich signal, curved DNA, and kinked DNA were identified in all three developmental datasets (Fig. 3C, Table 3). Of all the MAR motifs, AT richness was highest in all the datasets with a percentage abundance of 92.18, 92.28, and 87.44% and the percentage abundance of ORI signals was 77.46, 77.08, and 68.53%, in SG 1, SG 5, and SG 7, respectively. These findings suggest that PSGs are transcriptionally more active in the 5^th^ instar larval stage. The curved DNA signals in the datasets were 19, 5.68, and 11.63% in SG 1, SG 5, and SG 7, respectively. The signals for kinked DNA were 12.35, 6.43, and 13.02%, in SG 1, SG 5, and SG 7, respectively. TG-rich signals were observed at 2.11, 1.98, and 1.49% in SG 1, SG 5, and SG 7, respectively. The topoisomerase II signals were 0.77, 0.46, and 0.57%, in SG 1, SG 5, and SG 7, respectively. The MAR recognition signatures occurred with a frequency of 9.39, 9.08, and 5.77% in SG 1, SG 5, and SG 7, respectively. Our data show for the first time that MAR association in PSGs during development is dependent on multiple factors. Both sequence and structure of the DNA play a role in the association of chromatin to the NuMat.

**Table 3:** MAR-Associated features. The MAR associated motifs were identified in the SG 1, SG 5, and SG 7 datasets using RSAT. The number of times the signal is detected for these motifs in each dataset is shown

S. No.	Motif Name	Motif Index	Sequence	SG 1	SG 5	SG 7
1	ORI Signal	m_1_	ATTA	12611	40445	48448
2	ORI Signal	m_2_	ATTTA	4934	16748	18480
3	ORI Signal	m_3_	ATTTTA	1670	5082	5623
4	TG Rich Signal	m_4_	TGTTTTG	75	307	386
5	TG Rich Signal	m_5_	TGTTTTTTG	37	48	36
6	TG Rich Signal	m_6_	TTTTGGGG	9	18	81
7	Curved DNA Signal	m_7_	AAAAn_7_AAAAn_7_AAAA	75	183	90
8	Curved DNA Signal	m_8_	TTTTn_7_TTTTn_7_TTTT	75	183	90
9	Curved DNA Signal	m_9_	TTTAAA	1161	4194	4202
10	Kinked DNA Signal	m_10_	Tan_3_TGn_3_CA	107	262	847
11	Kinked DNA Signal	m_11_	TAn_3_CAn_3_TG	589	694	3796
12	Kinked DNA Signal	m_12_	TGn_3_TAn_3_CA	72	160	215
13	Kinked DNA Signal	m_13_	TGn_3_ CA n_3_TA	107	262	847
14	Kinked DNA Signal	m_14_	CA n_3_TAn_3_TG	111	470	468
15	Kinked DNA Signal	m_15_	CA n_3_TGn_3_TA	589	694	3796
16	mtopo-II Signal	m_16_	RnYnnCnnGYnGKTnYnY	18	27	97
17	dtopo-II Signal	m_17_	GTnWAYATTnATnnR	11	30	30
18	AT Rich Signal	m_18_	WWWWWW	22009	37915	77404
19	MRS	MRS		160	560	640

### Identification of genomic and gene associated functions of MARs

**Fig. 4:**
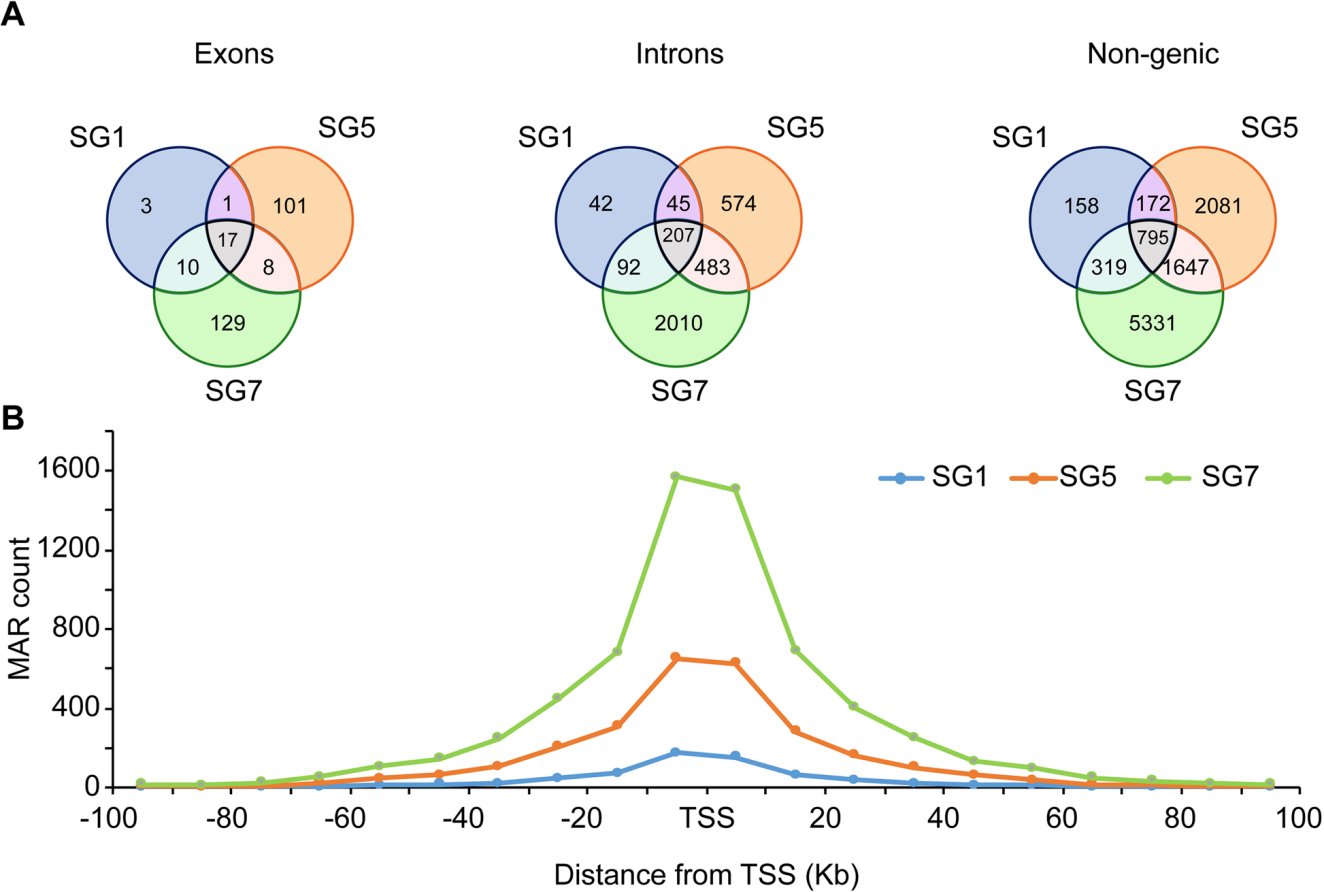
MAR localization in Genomic Elements: The MARs obtained on day 1, day 5 and day 7 were checked for their distribution in *B. mori* genome. **A** MARs present in exons, introns and non-genic regions (from left to right) in the three datasets **B** Distance of MARs from transcription start site (in the -100 kb to + 100 kb region) is plotted against the MAR count found in these regions among the three datasets

MARs have been identified in intronic, exonic, and non-genic regions [64]. The number of introns, exons, and non-genic regions associated with the NuMat was determined in the datasets of SG 1, SG 5, and SG 7 by matching the datasets to the gene models and gene lists and comparing to the individual datasets. 1.11, 37.54, and 47.95% of the identified exons of the three datasets were found to be unique to SG 1, SG 5, and SG 7, respectively while 6.31% of the exons were common to the three datasets. 1.21, 16.62, and 58.21% of the identified introns of the three datasets were found to be unique to SG 1, SG 5, and SG 7, respectively while 5.99% of the introns were common to the three datasets. Of the total 10,503 non-genic regions identified, 7.56% were common among the three datasets while 1.50, 19.81, and 50.75% unique non-genic regions were found in SG 1, SG 5, and SG 7 (Fig. 4A). MARs were enriched in non-genic regions followed by intronic and exonic regions. Significant differences in the number of non-genic, intronic, and exonic MARs were found between the SG 1, SG 5, and SG 7. The analysis confirmed the increasing association of MARs from day 1 to day 7 of 5^th^ instar development. The enrichment of MARs in the close proximity of the transcription start site (TSS) validated their importance in the transcriptional regulation of genes [18]. The region spanning the − 100 kb to + 100 kb region from the TSS was examined for the presence of MARs. The results showed that the highest peaks in all three datasets (SG 1, SG 5, and SG 7) were observed in the region from − 10 to + 10 kb flanking the TSS (Fig. 4B).

**Fig. 5:**
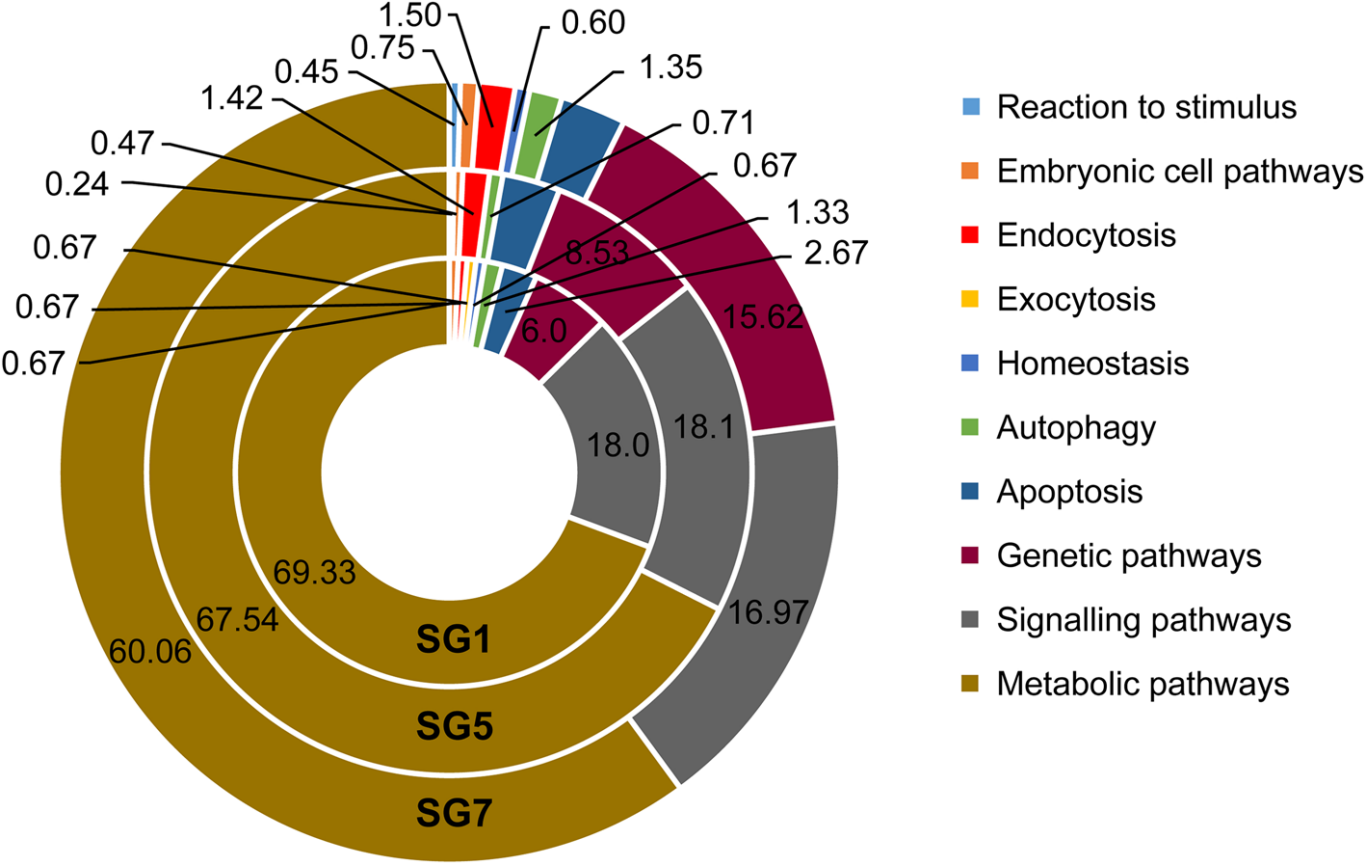
Biological Pathways: The gene list of the annotated reference genome from Silkbase was used to annotate the datasets through gene IDs which were matched against those of *Bombyx mori* database in SGID. Further, the genes found to be associated with the MAR datasets were used to draw a comparison of various biological pathways associated with these genes. The shift in the number of genes associated with the biological pathways during development are possibly due to the chromatin dynamics and MARs responsible for chromatin loop formation

A comparison of the three datasets based on the association between the MAR and the genes involved in various biological pathways revealed that the most significant pathways in the three datasets were the key biological pathways-the metabolic pathways, the genetic pathways, and the signalling pathways. An increased association was observed between the MAR and the genes involved in apoptosis, ribosome biogenesis, transcription, and DNA repair associated pathways from day 1 to day 7 (Fig. 5). The pathways associated with transcription and DNA repair increased significantly with developmental progression. These results correlate with the increased secretion of silk protein from PSGs.

### Dynamic association of FIBH and Br- c regions with the NuMat

**Fig. 6:**
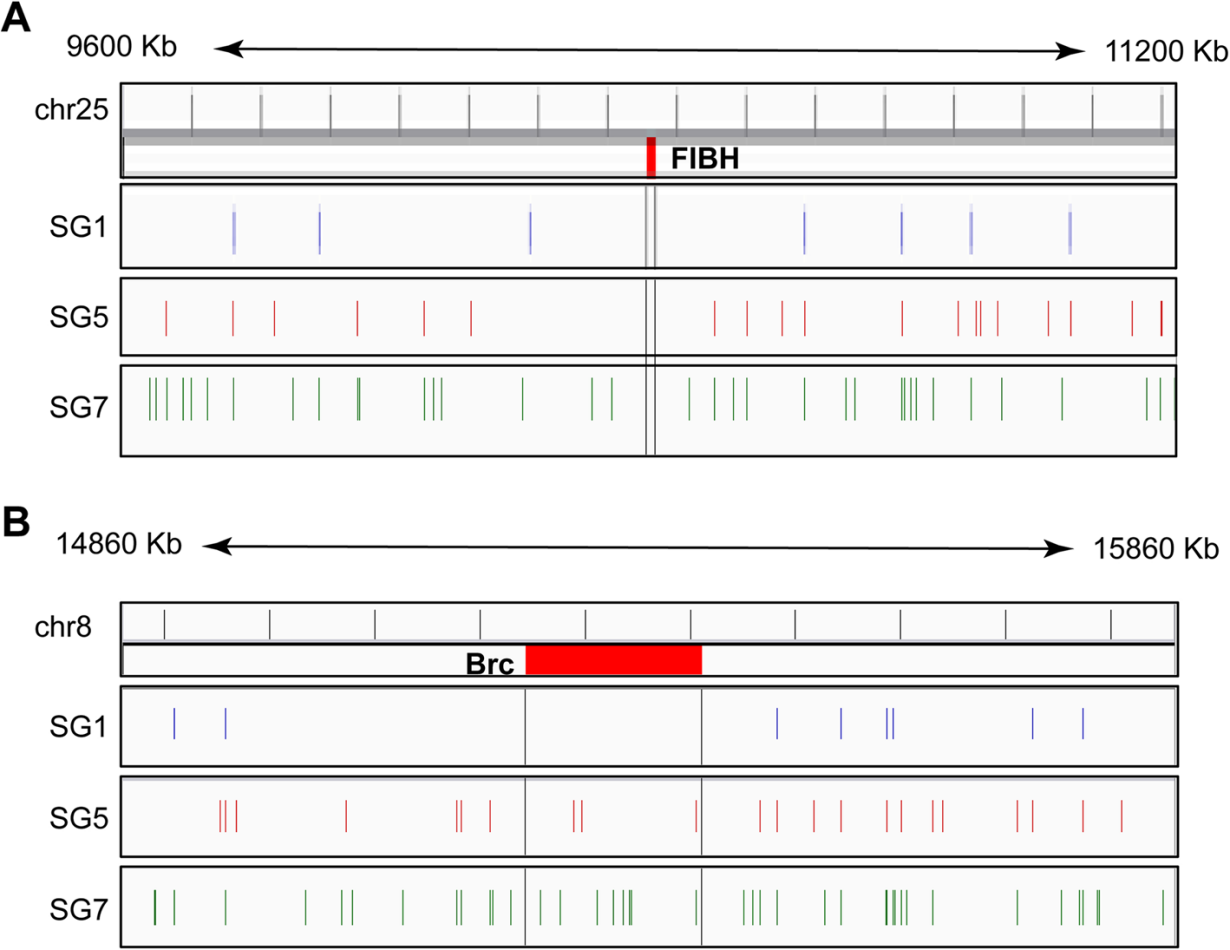
Visualization of MARs flanking FIBH and Br-c across the datasets. **A** The 1600Kb flanking region of FIBH gene (Chr 25). **B** The 1000Kb flanking region of Br-c gene (Chr 8)

The association of genes with the NuMat is important for gene regulation [65]. To understand the dynamics of the fibroin gene regulation, the 1600 kb (800 kb upstream and 800 kb downstream) region flanking FIBH gene was examined for MARs in SG 1, SG 5, and SG 7. The MARs were found to differ in number and position among the three datasets (Fig. 6A). The distribution of MARs in the flanking region of FIBH on the three developmental time point was different. Forty-five MARs were found in this region, of which forty-two MARs were found to be developmentally different. Results show that when development progressed from SG 1 to SG 5, and SG 5 to SG 7, new MARs were produced indicating the formation of new chromatin domains. There were 1, 8, and 24 unique MARs in SG 1, SG 5, and SG 7 datasets, respectively while all three datasets shared 3 common MARs in the 1600 kb FIBH flanking region.

Previous work from our lab showed that the broad-complex negatively regulates fibroin gene expression [66]. This was further supported by a recent study that showed that the overexpression of BmBr-c Z2 in the transgenic lines of *B. mori* results in the decrease levels of FIBH, Fib-l and P25 silk proteins. This mechanism was shown to be connected with the juvenile hormone pathway which is linked to the expression of its TFs, Bmdimm and BmKr-h1 [67]. The expression of Bmdimm was shown to positively regulate FIBH which correlates with the effect of Br-c expression on the regulation of FIBH expression [68]. Therefore, the 1000 bp flanking regions of Br-c gene were examined for MAR association. The number of MARs increased from day 1 to day 7 (Fig. 6B). MARs were observed within the gene region of Br-c on day 5 and day 7. The number of MARs associated with the matrix of Br-c increased significantly from SG1 to SG7. Gene silencing has been shown to occur when MARs are located within a gene (NuMat attachment-induced silencing) [69]. Our data suggest that the MARs found in the coding regions of Br-c may be involved in the down-regulation of the broad-complex which in turn increase the FIBH expression. This is consistent with studies that showed ectopic expression of Br-c Z2 inhibited FIBH expression [67].

## Discussion

The NuMat helps in the maintenance of the higher-order chromatin structure and stability [70, 71]. MARs bind and regulate chromatin regions and allow temporal and spatial regulation of genes. Not many studies have analysed the MAR sequences at the genome level. Our study characterized for the first time, tissue-specific genome-wide MARs during 5^th^ instar larval development in *B. mori*, to understand the dynamics associated with chromatin architecture in PSGs. Sequences were found to be enriched at the 200–400 bp range and TEM imaging showed the inner architecture of the PSG NuMat. Both the size of MARs associated with the NuMat and the architecture matched previous studies. The median MAR length enrichment in humans is 596 bp [18], and in *Drosophila* is 400 bp [13].

MARs of the 5^th^ instar PSGs showed common and variable regions across the genome. The common regions are important for the constitutive function of the genome, while variable regions are essential for the dynamic expression of genes during development. Inter-MAR associations aid in the formation of transcriptionally active chromatin loops and regulate gene expression [72]. They enable chromatin loop formation which dictates the initiation and regulation of transcriptional mechanisms [73–75]. The NuMat-associated TFs that bind at the MARs and the actively transcribed genes often coexist with the same transcriptional machinery [76, 77]. Hence the chromatin loops are important for chromatin function. The average loop size of chromatin is in the range of 20–200 kb in HCT116 cell line and HeLa cells [78, 79]. In silkworms, Li et al. demonstrated that chromatin loop size decreased when development progressed from day 1 to day 7 potentially resulting in significant differences in expressed transcripts from the 4th moult until the wandering stage of PSGs. The average chromatin loop size has been shown to vary from 40 kb to 254 kb in different days of 5^th^ instar development. The size of the MAR anchored loops appears to influence the expression efficiency of the genes. Therefore, a decrease in loop size towards day 7 in PSGs likely promotes gene expression. Future experimental studies can be carried out to verify the effect of chromatin loop formation on gene expression.

To determine the MAR enrichment pattern in the genome, a chromosome-wise MAR distribution analysis was performed and the number of MARs per chromosome in SG 1, SG 5, and SG 7 were determined. The enrichment of MARs was checked for correlation against gene density in each chromosome and a weak positive correlation was seen between the MAR count in each chromosome and its gene density. This corroborates that MARs are relatively enriched in gene-rich regions of the genome.

In a follow up analysis to the current study, we selected specific genes from the protein expression data of previous work from our lab [80], which were differentially expressed, and found that the MARs found within these genes were dynamic in their spatio-temporal association in the NuMat. The variation in the location of the MARs and the occurrence of new MARs with developmental progression was observed among day 1 (SG 1), day 5 (SG 5) and day 7 (SG 7) PSG MAR datasets (Supplementary Table 2). These MARs may be involved in the structural or functional roles during development in the PSGs of the 5^th^ instar larval stage. MARs were also found to be present in the intronic regions. Similar findings were reported in a study on the MAR regions and their correlation with gene expression levels in *Arabidopsis thaliana,* where the expression data from previous studies was used to compare the expression levels of MAR containing genes and genes lacking MARs. It was found that the genes containing MARs had lower expression levels as they were down-regulated due to higher association with regulatory elements. Intragenic/ intronic MARs were said to cause tissue and organ specific regulation of gene expression. The differential expression of MAR containing genes and genes without MARs was explored during different stages in 2 different tissues (root and flower) and it was found that the MAR containing genes were involved in gene regulatory functions.TF families were enriched in S/MARs and a synergistic effect was observed for tissue and organ specific expression of TF genes and the presence of intragenic S/MARs [81].

Previous studies found an association of repeats to be correlated with gene expression [82]. TEs are known to be associated with the NuMat. They are known to influence genome plasticity, genome architecture, and chromatin loop formation [44, 45]. They often carry embedded TF binding sites and therefore contribute to transcription and gene expression [83]. L1Bm repeats were found in the intronic region along with an AT enriched modulator of fibroin [46, 47]. L1Bm was also found to be an integral part of Bmmar1, a MAR identified in the 62 kb upstream region of fibroin [30]. Its association with the NuMat was further validated by its presence throughout the 5^th^ instar development. The STRs, TTAGG, and CCTAA, enriched in the datasets are known telomeric repeats of *B. mori* and have many LTR repeats integrated into them which include L1Bm [32, 84]. Moreover, telomeric repeats are associated with the NuMat [51]. Other TEs like *gypsy, jockey,* and *roo* involved in establishing chromatin boundaries were also found to be associated with the NuMat [13, 14, 85]. The LTR transposon, Ty1 associated with certain regions of chromatin and an important player in genome organisation, along with R1Bmks, a non-LTR transposon of *B. mori*, was also found to be associated with the NuMat. STRs also play a role in gene expression [86] and enrichment of repeat sequences was found in all three datasets. SSR discovery was carried out using MISA web, a web server for microsatellite prediction. The default parameters i.e., minimum number of repetitions set as 5 for their detection by the algorithm and maximum length of sequence between two SSRs to register as compound SSR set as 100 were used for this analysis. The compound sequences for tetra, penta and hexa nucleotides were also included as individual signals.

MAR sequence data sets in the current study on all 3 days showed AT base enrichment as observed in previous studies [13, 87]. In addition, we identified a wide range of motifs that were associated with the NuMat [88]. While AT richness is often associated with flexibility, certain recognition sites for protein-DNA binding and narrowing of the minor groove [89]; curved and kinked DNA motif signals are identified by their overall shape and are bound by proteins [90, 91]; topoisomerase II motif signals are important for supercoiling as well as preventing rotation of the double helix [92]; and ORI signals are strongly associated with the NuMat and execute or prevent the replication process and are hence important in gene regulation [93]. The MAR recognition signatures which are two proximal degenerate sequences are common to most MARs identified and were also found in all three datasets [94]. The three most abundantly overrepresented motifs that we identified were putative TF binding sites. These signature motifs of day 1, day 5, and day 7 were found to be different. The variations in the three most abundantly overrepresented motif sequences among the three datasets indirectly convey the change in the precedence of the genes transcribed during development and the dynamic role of MARs and their influence in gene expression and regulation. Future work focusing on these motifs will help understand their relevance across other genomes.

The association of the matrix with intergenic regions is involved in protecting genes from being silenced [95]. MARs present in the exonic regions may be involved in transcription [13]. Although not much is known about MARs present in the intronic regions [96], they mainly occur at the flanks of transcribed regions, in 5′-introns and telomeres [97]. To address the genomic context of MARs, their presence in different genomic regions was determined. The results portrayed enrichment of MARs in the order: non-genic>introns>exons. MAR distance from the TSS affects the transcription of genes [98]. The enrichment of MARs throughout the -100 kb to + 100 kb region flanking the TSS was established with many MARs enriched proximal to the TSS. The enrichment of intergenic MARs proximal to the TSS may influence the transcription of genes downstream to them, resulting in the enhanced expression of genes in PSGs from day 1 to day 7. The MAR association with genomic elements was also determined similarly in other studies [13, 18]. The association of MAR with genomic elements was also found in plant species such as *Arabidopsis* [15, 81].

To further understand the functions of genes regulated by these MARs, the biological pathways of the MAR-associated genes were explored. We found that the metabolic pathway genes were most abundantly associated with the NuMat at all the three-time points of 5^th^ instar larval development. These results confirm that PSGs express more genes involved in metabolic control as they progress towards the wandering stage [80, 99].

The current study identified common and variable MARs flanking the FIBH gene in PSG of 5^th^ instar larvae which showed day-specific variations. Many MAR regions were identified for the first time flanking the FIBH gene. This is consistent with previous literature which shows that actively transcribed genes are associated with the NuMat [65]. Since the broad complex was shown to negatively regulate FIBH expression [66], the Br-c gene was analysed for MARs. Though matrix attachment in intergenic regions can protect genes from being silenced, the presence of MARs within genes correlates with gene silencing [69, 95]. Br-c showed presence of MARs in the gene region which could contribute towards the downregulation of the genes on day 5 and day 7 of the 5^th^ instar larval stage. The identified MARs likely downregulate the expression of broad complex which in turn upregulates FIBH gene expression on day 5 and day 7 of the 5^th^ instar stage.

Our study is the first to report the developmental analysis of genome-wide MAR sequencing data in the PSGs of *B. mori*. The results of the present analysis show that the organization of the silk gland genome is under the dynamic control of the NuMat. These elements and their association to PSG NuMat can be linked directly or indirectly in the regulation of FIBH expression.

## Conclusions

The retention of nuclear DNA in NuMat was found to be 4.33, 9.41 and 13.24% in the PSGs of day 1, day 5 and day 7 respectively. MARs show dynamic nature and are found to vary in their attachment to NuMat. Their number of varied among day 1, day 5 and day 7. The number of MARs increased from day 1 to day 7 which caused the decrease in chromatin loop length. The number of MARs showed positive correlation with the gene density on the chromosomes. TE, L1Bm, found in the 62 kb upstream region of FIBH, predicted to influence its expression was found in all the 3 days of 5^th^ instar development. MARs were found abundantly in non-genic regions when compared to introns and exons. The MARs present within the gene Br-c are predicted to inhibit broad complex expression thereby increasing the expression of FIBH. The MARs identified close to the TSS may be associated with transcriptional regulation. Analysis of MAR DNA associated genes showed their role in regulation of transcription and DNA-repair.

This study is the first tissue-specific genome-wide study that explores MAR dynamics and their effect on chromatin organization during 5^th^ instar development in PSGs and predicts that FIBH gene expression is regulated by MARs associated to different chromatin regions.

## Methods

### Isolation and quantification of nuclear and MAR DNA

Bivoltine, double hybrid, CSR2 X CSR4, *B. mori* 4th moult larvae were collected from the Department of Sericulture, Srikakulam, Government of Andhra Pradesh. 100 Larvae were fed fresh V1 mulberry leaves from day 1 to day 7 (wandering stage) of the 5^th^ instar stage. PSGs were dissected from 5th instar larvae on day 1, day 5, and day 7. PSGs were pooled (day1 (*n* = 30), day 5 (*n* = 15), and day 7(*n* = 10)) from a single rearing, and 1 g tissue from each day was homogenized in nuclear isolation buffer (5 times the volume of the weight of the tissue) and processed for nuclei and NuMat isolation by following the standard protocol for isolation through nuclease digestion and salt extraction [13] (Supplementary Fig. 2, Supplementary Fig. 3).

The nuclear and MAR pellets were further processed for DNA isolation with HiPurA™ Genomic DNA Purification Kit. Quantification of nuclear and NuMat PSG DNA on day 1, day 5, and day 7 was carried out by UV spectrophotometry (Fig. 1A, Supplementary Table 3). Agarose gel electrophoresis (Fig. 1B) and Tapestation profiling using Agilent 2200 TapeStation, were performed to check the enrichment of MARs and their fragment size distribution in the libraries respectively (Supplementary Fig. 4). The MAR DNA, consists of small but varying size fragments and therefore appears as a smear instead of a clear band on the agarose gel (Fig. 1B).

### Transmission electron microscopy (TEM) analysis

Transmission electron microscopy was performed to confirm the isolation of the NuMat by nuclease digestion and salt extraction. Sections of day 5 PSGs were made by fixing samples in 2.5% glutaraldehyde in 0.1 M phosphate buffer (pH 7.2) for 24 h at 4^o^ C, and washing with PBS (phosphate buffer saline) for 4 times (each wash for 1 h). Post fixation was carried out in aqueous osmium tetroxide for 3 h. Washes were performed 6 times (each for 1 min) with deionized distilled water. The samples were dehydrated in a series of graded alcohols, infiltrated, and embedded in Araldite resin. Incubation was done at 80^o^ C for 72 h for complete polymerization. Ultra-thin (60 nm) sections were made with a glass knife on ultra-microtome (Leica Ultra cut UCT-GA-D/E-1/00), mounted on copper grids, and stained with saturated aqueous uranyl acetate (UA) and counterstained with Reynolds lead citrate (LC) [36]. JEOL JEM-2100 Electron Microscope with GATAN ULTRASCAN camera, charged coupled detector and digital micrograph software was used for TEM Imaging. TEM imaging was carried out at Ruska Labs, Acharya N. G. Ranga Agricultural University, Hyderabad, Telangana.

### Library preparation and sequencing

MAR DNA sequencing libraries were prepared with Illumina-compatible NEXTflex ChIPSeq Library Prep Kit (Bio Scientific, Austin, TX, U.S.A.) at the Genotypic Technology Pvt. Ltd., Bangalore, India. DNA was sheared using a Covaris S220 system (Covaris, Woburn, Massachusetts, USA) and the fragmented DNA was purified using magnetic beads and subjected to end repair. Adapter ligation was carried out to the end-repaired DNA after 3′ adenylation. The adapter-ligated DNA was purified with JetSeq magnetic beads and then amplified for 8 cycles (denaturation at 98^o^ C for 2 min, cycling [98^o^ C for 30 s, 65^o^ C for 30 s, and 72^o^ C for 1 min] and a final extension at 72^o^ C for 4 min). The final PCR product was purified with JetSeq beads (Bio, # 68031), followed by a library-quality control check. The sequencing library was initially quantified by Qubit (Thermo Fisher Scientific, MA, U.S.A.) (Supplementary Table 4).

### MAR-Seq data analysis

The raw sequenced data obtained from the Illumina sequencing platform as single-end sequencing were labelled SG 1, SG 5, and SG 7 (day1, day 5, and day 7), respectively, and the quality control for each of these three datasets was carried out using FastQC tool v1.1 (https://www.bioinformatics.babraham.ac.uk/projects/fastqc/). The adapters were trimmed using cutadapt (Version 1.16) [37].

The MAR library reads of SG 1, SG 5, and SG 7 were aligned to the *B. mori* genome (Silk Base Version 2017.4.21) [38]. Mapping was performed using a BOWTIE2 wrapper available on the galaxy platform [39]. The wrapper internally uses bowtie2 (version 2.3.4.1) and samtools (version 1.9) [40, 41].

The mapped reads were further analysed for sequencing depth using the plotCoverage tool (Galaxy Version 3.3.200.0) which utilises deeptools (Version 3.3.2) and samtools (Version 1.9).

The MAR peaks were identified using MACS2 callpeak from the galaxy platform which internally uses MACS2 (Version 2.1.1.20160309) and r-base (Version 3.4). The identified MAR peaks were merged for the overlapping intervals and bookended intervals into a single interval using MergeBED from bedtools (Version 2.29.2). The merged BED files were used for determining the distribution of MARs in different chromosomes and the size of the MARs across the three datasets (SG1, SG 5 and SG 7). The average loop length was determined by calculating the distance between MARs. Analysis of unique and common MAR regions in SG 1, SG 5, and SG 7 was performed with bedtools intersect command (Version 2.29.0).

### MAR annotation and biological pathway analysis

The ‘bedops/closest features’ tool was used for annotating the MARs. The merged peaks of SG 1, SG 5, and SG 7 datasets in BED format and the *B. mori* reference genome (annotated genome version 2017.4.21 from Silkbase) were used for annotating the MAR peaks. The intronic, exonic, and non-genic regions along with the distances of MARs from the transcription start site (TSS) were determined. The MAR-associated genes were also identified in the analysis and were used to identify associated pathways utilizing the ‘pathways’ tool from the silkworm genome informatics database [42].

### Analysis of MAR associated features in the datasets

The merged peaks were used as input and the various motifs found to be associated with MARs such as ORI Signals (ATTA, ATTTA, ATTTTA), topoisomerase II signals (RnYnnGYnGKTnYnY, GTnWAYATTnATnnR), TG Rich Signal (TGTTTTG, TGTTTTTTG, TTTTGGGG), AT-rich signal (WWWWWW), Curved DNA (AAAAn_7_AAAAn_7_AAAA, TTTTn_7_TTTTn_7_TTTT, TTTAAA) and Kinked DNA (TAn_3_TGn_3_CA, TAn_3_CAn_3_TG, TGn_3_TAn_3_CA, TGn_3_CAn_3_TA, CAn_3_TAn_3_TG, CAn_3_TGn_3_TA) were identified in the three developmental datasets with the help of Regulatory Sequence analysis tools (RSAT Metazoa) [43, 44]. MAR Recognition Signatures (MRS) (bipartite sequence element that consists of two individual sequences of 8 (AATAAYAA) and 16 bp (AWWRTAANNWWGNNNC) within a 200 bp distance from each other) were identified in the datasets using EMBOSS marscan from galaxy server which internally uses emboss (Version 5.0.0) and Perl (Version 5.26).

### Identification of repeats

The STR repeats were identified for the enriched MAR peaks in the three datasets. The MAR peaks were converted to FASTA format and used for extracting Short Tandem Repeats (STRs) of di-, tri-, tetra-, penta- and hexanucleotides. MISA-web (Version 2.1, updated: 2020-08-25) tool was used for finding tandem repeats [45]. The default parameters i.e., minimum number of repetitions set as 5 for their detection by the algorithm and maximum length of sequence between two SSRs to register as compound SSR set as 100 were used for this analysis. The analysis was carried out to identify the signals of the repeat sequences picked throughout the datasets and not in accordance with the number of times the repeat occurs in a specific location in the genome. The compound sequences for tetra, penta and hexa-nucleotides were also included as individual signals.

The FASTA sequences of TEs known to be associated with the NuMat were extracted from the NCBI database and aligned against the merged BED files of the datasets SG 1, SG 5, and SG 7 using the BLAST2 tool to find their distribution in the developmental datasets. The hits with > = 90% percentage identity with > = 100 bp sequence length were considered. Other TEs associated with the genome were identified using rmblast of RepeatMasker (http://www.repeatmasker.org) with the closest available repbase library which was of *D. melanogaster.*

### Developmental analysis of FIBH and Br-c flanking MARs

The MAR peaks were loaded into the integrated genome viewer (Version 2.6.3) and MARs distribution was visualized, among the developmental datasets: SG 1, SG 5, and SG 7, in the flanking regions of the genes FIBH and Br-c.

### Statistical analysis

Student’s t-test: 2-sample assuming unequal variances, was used to determine the significance of variation in the nuclear and MAR DNA isolated from PSGs of 5^th^ instar larval stage on day 1, day 5 and day 7. *p* ≤ 0.5 was considered as significant; *p* > 0.5 was considered as not significant. The experiment was conducted five times each for isolation and estimation of nuclear and MAR DNA respectively. Pooled samples of PSG tissues of day 1, day 5 and day 7 (1 g tissue of PSGs for each day) were used in the experiments.

## Supplementary Information

**Additional file 1: Supplementary Fig. 1.** Scatter plot of MAR Count against Gene Density among the three datasets. **Supplementary Fig. 2.** NuMat preparation. The steps involved in the NuMat extraction and MAR DNA isolation are depicted in the flowchart. **Supplementary Fig. 3.** MAR DNA Isolation from day 1, day 5 and day 5 PSGs. The SG 1, SG 5 and SG 7 lanes represent MAR DNA isolated from day 1, day 5 and day 7 PSGs respectively. The lanes represented by ‘M’ indicate 1 kb ladder run alongside the MAR DNA samples. Lane ‘N’ refers to nuclear DNA isolated from day 5 PSGs. **Supplementary Fig. 4.** Determination of Size Distribution of MAR DNA isolated from 5th instar PSGs. Tapestation profiling showing (A) 25–1500 bp ladder and enriched size range in (B) day 1 MAR DNA (C) day 5 MAR DNA) and (D) day 7 MAR DNA isolations. **Supplementary Table 1.** Read count statistics of raw sequenced data. The total number of reads, the percentage of unique sequences obtained, and the percentage GC content from the raw sequenced data is given. **Supplementary Table 2.** MARs associated with differentially expressed genes. Examples of differentially expressed genes with MARs identified within the genes by BLAST analysis of the three PSG 5th instar MAR developmental datasets (day 1, day 5 and day 7) are shown. The MAR regions in bold refer to those present in the intronic regions of the gene. **Supplementary Table 3.** Estimation of nuclei and NuMat DNA in day 1, day 5, and day 7 posterior silk glands of *B. mori.* The MAR DNA isolated from day 1, day 5, and day 7 PSGs were quantified and the average concentration along with standard error values are provided. **Supplementary Table 4.** Library Concentration estimation using Qubit. Quantification of day 1, day 5, and day 7 PSGs from 5th instar *B.mori* larvae was performed using Qubit and the barcode sequences used in the library preparation are provided.

## Data Availability

The data described in the current study can be freely and openly accessed on the National Centre for Biological Information (NCBI), Sequence Read Archive (SRA) database under the bio project number PRJNA555810 under the accessions and links: SRX6480237 (https://www.ncbi.nlm.nih.gov/sra/SRX6480237[accn]) for SG 1, SRX6480247 (https://www.ncbi.nlm.nih.gov/sra/SRX6480247[accn]) for SG 5 and SRX6480293 (https://www.ncbi.nlm.nih.gov/sra/SRX6480293[accn]) for SG 7 respectively.

## Abbreviations

NuMat: Nuclear Matrix
MAR: Matrix Attachment Region
LTR: Long Terminal Repeat
ASG: Anterior Silk Gland
MSG: Middle Silk Gland
PSG: Posterior Silk Gland
TEM: Transmission Electron Microscopy
NL: Nuclear Lamina
Nu: Nucleolus
dCh: condensed chromatin
lCh: open chromatin
SG 1: day 1 MAR dataset of 5^th^ instar PSGs
SG 5: day 5 MAR dataset of 5^th^ instar PSGs
SG 7: day 7 MAR dataset of 5^th^ instar PSGs
STR: Simple Tandem Repeat
TE: Transposable Element
LINEs: Long Interspersed Nuclear Element
TF: Transcription Factor
PBS: Phosphate Buffer Saline
UA: Uranly Acetate
LC: Lead Citrate
TSS: Transcription Start Site
RSAT: Regulatory Sequence Analysis Tools
MRS: MAR Recognition Signature
