# Supplementary material for: Dynamics of nuclear matrix attachment regions during 5th instar posterior silk gland development in Bombyx mori
Source: BMC Genomics. 2022 Mar 31;23:247. doi: 10.1186/s12864-022-08446-3 (PMC8973518; doi:10.1186/s12864-022-08446-3)
Supplement: Supplementary file 1 — Additional file 1: Supplementary Fig. 1. Scatter plot of MAR Count against Gene Density among the three datasets. Supplementary Fig. 2. NuMat preparation. The steps involved in the NuMat extraction and MAR DNA isolation are depicted in the flowchart. Supplementary Fig. 3. MAR DNA Isolation from day 1, day 5 and day 5 PSGs. The SG 1, SG 5 and SG 7 lanes represent MAR DNA isolated from day 1, day 5 and day 7 PSGs respectively. The lanes represented by ‘M’ indicate 1 kb ladder run alongside the MAR DNA samples. Lane ‘N’ refers to nuclear DNA isolated from day 5 PSGs. Supplementary Fig. 4. Determination of Size Distribution of MAR DNA isolated from 5th instar PSGs. Tapestation profiling showing (A) 25–1500 bp ladder and enriched size range in (B) day 1 MAR DNA (C) day 5 MAR DNA) and (D) day 7 MAR DNA isolations. Supplementary Table 1. Read count statistics of raw sequenced data. The total number of reads, the percentage of unique sequences obtained, and the percentage GC content from the raw sequenced data is given. Supplementary Table 2. MARs associated with differentially expressed genes. Examples of differentially expressed genes with MARs identified within the genes by BLAST analysis of the three PSG 5th instar MAR developmental datasets (day 1, day 5 and day 7) are shown. The MAR regions in bold refer to those present in the intronic regions of the gene. Supplementary Table 3. Estimation of nuclei and NuMat DNA in day 1, day 5, and day 7 posterior silk glands of B. mori. The MAR DNA isolated from day 1, day 5, and day 7 PSGs were quantified and the average concentration along with standard error values are provided. Supplementary Table 4. Library Concentration estimation using Qubit. Quantification of day 1, day 5, and day 7 PSGs from 5th instar B.mori larvae was performed using Qubit and the barcode sequences used in the library preparation are provided. [file 12864_2022_8446_MOESM1_ESM.pdf]

# Supplementary Data

| Details of the library | SG 1 | SG 5 | SG 7 |
|---|---|---|---|
| **Total Reads** | 26594084 | 30304527 | 44725766 |
| **Unique sequences** | 75.38 % | 78.95 % | 70.24% |
| **% GC content** | 41 | 38 | 41 |

**Supplementary Table 1.**

**Read count statistics of raw sequenced data**. The total number of reads, the percentage of unique sequences obtained and the percentage GC content from the raw sequenced data is given.

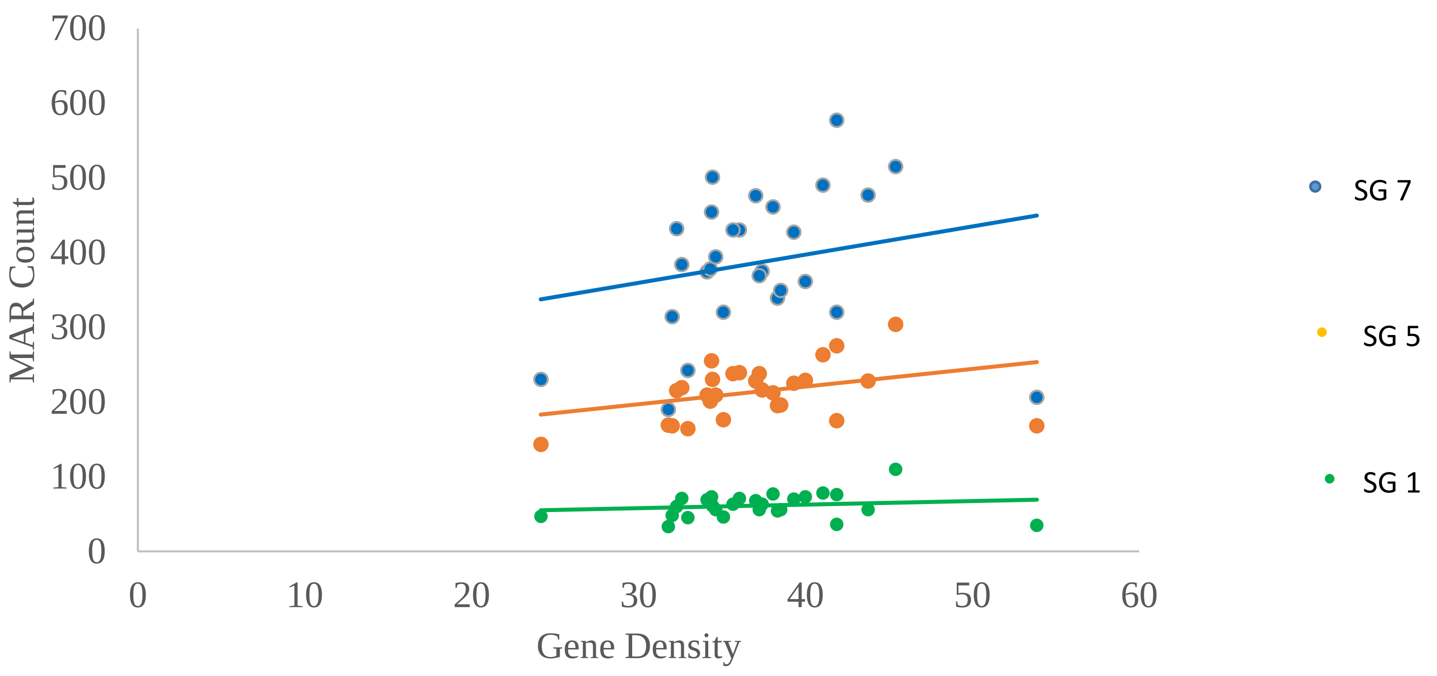


**Supplementary Figure 1.**

**Scatter plot** of MAR Count against Gene Density among the three datasets.

| S. No. | Gene ID | Gene | Protein | Predicted MAR | MARs found in datasets | | |
|---|---|---|---|---|---|---|---|
| | | | | | SG 1 | SG 5 | SG 7 |
| 1 | 692737 | Cypa | (Cyclophilin like protein) cyclophilin A | 800 | No Match | No Match | No Match |
| 2 | 100101186 | LOC100101186 | Abnormal wing disc like protein | 510 | No Match | No Match | No Match |
| 3 | 778467 | **LOC778467** | Fructose 1,6-bisphosphate aldolase | 2700;6700;10600;23800 | 3989-4084; 4483-4691; 5108-6177; 8013-10088; 10493-11961; 13141-15089; 15747-15904; 17493-17741; 18003-18155; 21636-22223; 24573-24895; 25316-26145; 26728-27203; 27659-27931; 28077-28259; 32932-33189 | 3989-4055; 5097-5491; 5574-5649; 5851-6186; 7085-7306; 8013-8395; 8532-8739; 8837-8877; 9706-10084; 13141-13402; 13781-14037; 4361-14448; 15748-15894; 17491-17745; 21650-21922; 22098-22188; 25324-26144; 26728-27191; 27647-27930; 28080-28256; 32928-33188 | 3989-4685; 5105-5833; 5866-6183; 8010-8058; 8139-8403; 8831-8877; 9706-10089; 11329-11518; 13142-13399; 13777-14031; 14331-14444; 15748-15904; 17494-17745; 18003-18095; 18352-18922; 21652-21922; 24517-24880; 25324-25581; 25867-26145; 26725-27182; 27664-27925; 28087-28256; 32933-33189 |

**Supplementary Table 2.**

**MARs associated with differentially expressed genes**. Examples of differentially expressed genes with MARs identified within the genes by BLAST analysis of the three PSG 5$^{th}$ instar MAR developmental datasets (day 1, day 5 and day 7) are shown. The MAR regions in bold refer to those present in the intronic regions of the gene.

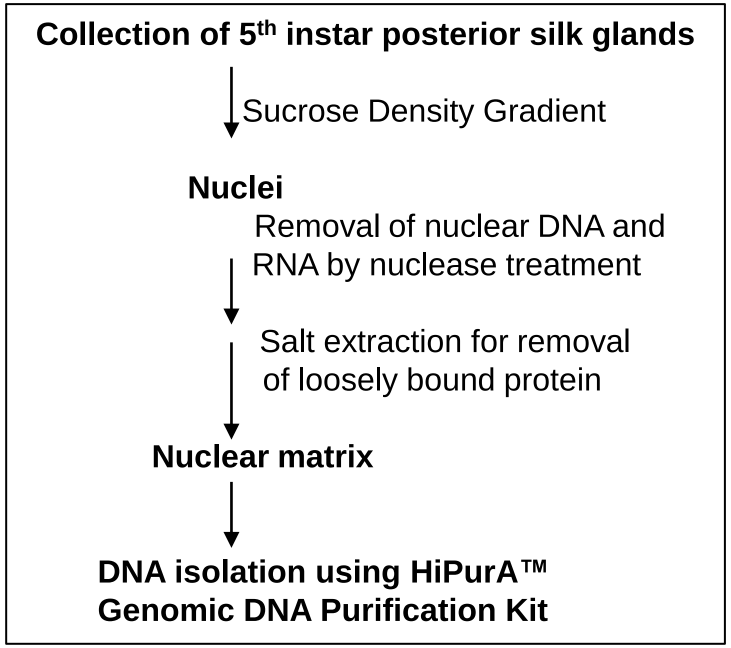


**Supplementary Figure 2.**

**NuMat preparation.** The steps involved in the NuMat extraction and MAR DNA isolation are depicted in the flowchart.

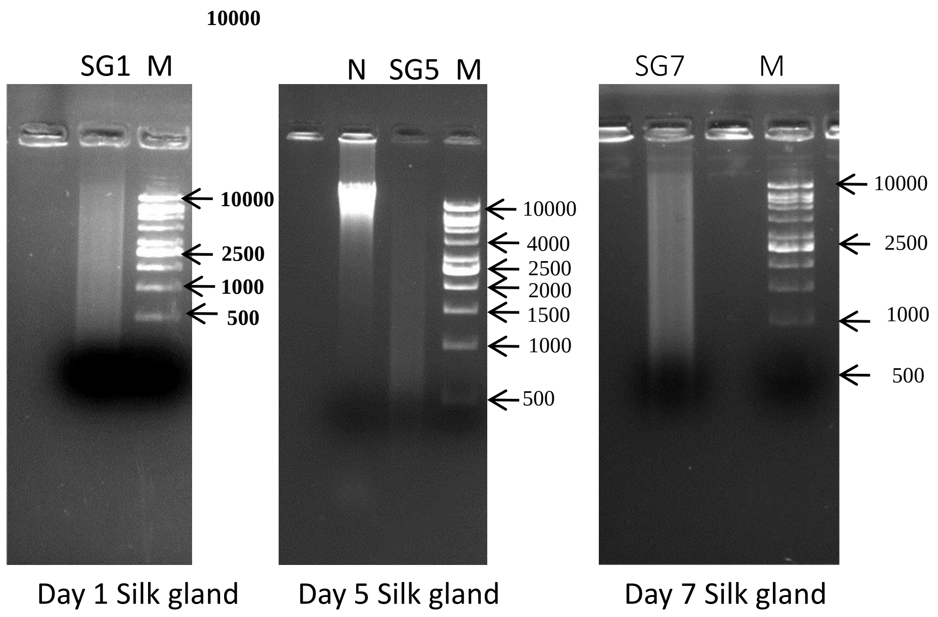


**Supplementary Figure 3.**

**MAR DNA Isolation from day 1, day 5 and day 5 PSGs.** The SG 1, SG 5 and SG 7 lanes represent MAR DNA isolated from day 1, day 5 and day 7 PSGs respectively. The lanes represented by ‘M’ indicate 1kb ladder run alongside the MAR DNA samples. Lane ‘N’ refers to nuclear DNA isolated from day 5 PSGs.

| Day | Nuclei concentration in µg (Average±Standard Error) | NuMat concentration in µg (Average±Standard Error) |
|---|---|---|
| 1 | 1360±38232303 | 59±13.85641 |
| 5 | 1700±251.1971 | 160±39.94997 |
| 7 | 1623.333±349.6188 | 215±50.22947 |

**Supplementary Table 3.**

**Estimation of nuclei and nuclear matrix DNA in day 1, day 5 and day 7 posterior silk glands of *B. mori.*** The MAR DNA isolated from day 1, day 5 and day 7 PSGs was quantified and the average concentration along with Standard error values are provided.

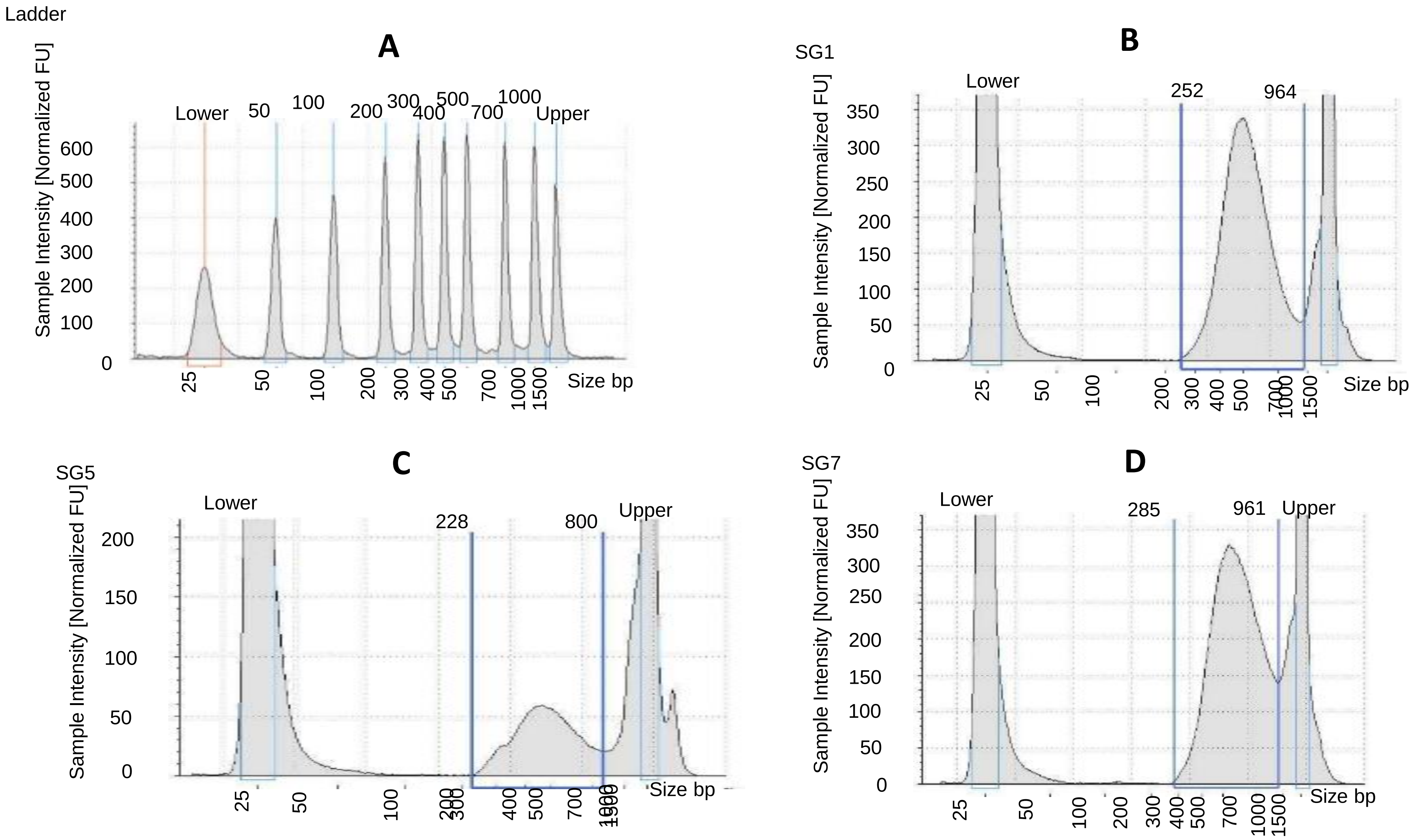


**Supplementary Figure 3.**

**Determination of Size Distribution of MAR DNA isolated from 5th instar PSGs.** Tapestation profiling showing (A) 25-1500 bp ladder and enriched size range in (B) day 1 MAR DNA (C) day 5 MAR DNA) and (D) day 7 MAR DNA isolations.

| S . No. | Sample ID | Qubit QC ng/µl | Volume (µl) | Yield (ng) | Netflex Barcode | Barcode sequence |
|---|---|---|---|---|---|---|
| 1 | SG 1 | 23.8 | 10 | 238 | NFBC21 | GTTTCG |
| 2 | SG 5 | 16.6 | 10 | 166 | NFBC22 | CGTACG |
| 3 | SG 7 | 20.8 | 10 | 208 | NFBC23 | GAGTGG |

**Supplementary Table 1.**

**Library Concentration estimation using Qubit**. Quantification of day 1, day 5 and day 7 PSGs from 5$^{th}$ instar B.mori larvae was performed using Qubit and the barcode sequences used in the library preparation are provided.
